# Discrimination against Homœopathists

**Published:** 1898-10

**Authors:** E. Rodney Fisk

**Affiliations:** Secretary; 484 Bedford Avenue, Brooklyn, N. Y.


### DISCRIMINATION AGAINST HOMŒOPATHISTS.

At a regular meeting of the Kings County Homœopathic Medical Society the following preamble and resolutions were unanimously adopted:

Whereas, The bigotry of the allopathic branch of the medical profession still exists and shows itself in discrimination by self-styled regulars against the appointment of homœopathists as surgeons or assistant surgeons in the army, navy, or marine hospital service of the United States; and

WHEREAS, The appointment of division surgeon, U. S. V., of Dr. M. O. Terry, who has served with distinguished success for two terms as Surgeon General N. G. N. Y., serves as a precedent and a proof that it is practicable for practitioners of Homœopathy to serve their country in their professional capacity.

*Resolved*, That the Homœopathic Medical Society of the County of Kings, State of New York, respectfully urge upon the House of Representatives, the Senate, and the President, the enactment of the joint resolution [Senate File 164, introduced by Senator Allen, of Nebraska], now before the Senate, to the effect that "Graduates in good standing of any medical college, regularly chartered under the laws of any State of the United States, and eligible to practice therein under the laws of such State, shall, on application, be entitled to examination for appointment in the medical corps of the army, navy, and marine hospital service of the United States, any statute or department ruling or regulation to the contrary notwithstanding."

*Resolved*, That a copy of this resolution be sent to every member of the Senate and of the House of Representatives, to President McKinley, to the Germantown Homœopathic Society, to Dr. A. B. Norton, Chairman of the Interstate Committee of the American Institute of Homœopathy, and the homœpathic journals of the country.

Respectfully yours,

E. RODNEY FISKE, M. D.,
*Secretary*.

484 BEDFORD AVENUE, BROOKLYN, N. Y.

